# Tissue eosinophilia induced by recombinant human interleukin-5 in the hamster cheek pouch membrane

**DOI:** 10.1155/S0962935195000536

**Published:** 1995-09

**Authors:** M. Minnicozzi, W. N. Durán, D. Kim, G. J. Gleich, J. Wagner, R. W. Egan

**Affiliations:** 1 Schering-Plough Research Institute Kenilworth NJ 07033 USA; 2 Department of Physiology UMDNJ-Graduate School of Biomedical Sciences Newark NJ 07103 USA; 3 UMDNJ-New Jersey Medical School Newark NJ 07103 USA; 4 Departments of Immunology and Internal Medicine Mayo Clinic and Mayo Foundation Rochester MN 55905 USA

## Abstract

Interleukin-5 (IL-5) is a cytokine that preferentially effects the development and function of eosinophils, and is considered important in the pathophysiology of allergic inflammation. In this study, we evaluated the ability of recombinant human IL-5 (rHu IL-5) to promote tissue eosinophilia and the importance of this eosinophilia to pathological alterations in vascular function. Repetitive subcutaneous administration for 18 days of rHu IL-5 resulted in a 7-fold increase in the number of eosinophils found in the ipsilateral hamster cheek pouch membrane. The contralateral cheek pouch membrane and peritoneum of these animals showed lesser but significant elevations in the number of eosinophils. In contrast, denatured rHu IL-5 did not elevate eosinophils in these tissues. Through the use of intravital microscopy and fluorometric analysis, rHu IL-5 treated hamster cheek pouch membranes were evaluated for alterations in microvascular permeability, using plasma clearance of FITC-dextran 150 as an index. Despite promoting a prominent tissue eosinophilia, the repetitive subcutaneous injections of rHu IL-5 did not alter the clearance of FITC-dextran 150. Topical application of rHu IL-5 to the cheek pouch, also, had no effect on the clearance of FITC-dextran 150. Immunofluorescence observations using an antibody to the granule protein, eosinophil peroxidase, indicated that the recruited cells had not degranulated. Our results support the importance of IL-5 in the recruitment of tissue eosinophils, but further stimulation is probably required to cause degranulation of these cells and the ensuing tissue damage.


Mediators of Inflammation 4, 331–338 (1995)

### Tissue eosinophilia induced by recombinant human interleukin-5 in the hamster cheek pouch membrane

M. Minnicozzi,[1,2,CA] W. N. Durán,[2,3] D. Kim,[3] G. J. Gleich,[4] J. Wagner[4] and R. W. Egan[1,3]

[1]Schering-Plough Research Institute, Kenilworth, NJ 07033, USA; [2]Department of Physiology, UMDNJ-Graduate School of Biomedical Sciences, Newark, NJ 07103, USA; [3]UMDNJ-New Jersey Medical School, Newark, NJ 07103, USA; [4]Departments of Immunology and Internal Medicine, Mayo Clinic and Mayo Foundation, Rochester, MN 55905, USA

[CA]Corresponding Author

**Interleukin-5 (IL-5) is a cytokine that preferentially effects the development and function of eosinophils, and is considered important in the pathophysiology of allergic inflammation. In this study, we evaluated the ability of recombinant human IL-5 (rHu IL-5) to promote tissue eosinophilia and the importance of this eosinophilia to pathological alterations in vascular function. Repetitive subcutaneous administration for 18 days of rHu IL-5 resulted in a 7-fold increase in the number of eosinophils found in the ipsilateral hamster cheek pouch membrane. The contralateral cheek pouch membrane and peritoneum of these animals showed lesser but significant elevations in the number of eosinophils. In contrast, denatured rHu IL-5 did not elevate eosinophils in these tissues. Through the use of intravital microscopy and fluorometric analysis, rHu IL-5 treated hamster cheek pouch membranes were evaluated for alterations in microvascular permeability, using plasma clearance of FITC-dextran 150 as an index. Despite promoting a prominent tissue eosinophilia, the repetitive subcutaneous injections of rHu IL-5 did not alter the clearance of FITC-dextran 150. Topical application of rHu IL-5 to the cheek pouch, also, had no effect on the clearance of FITC-dextran 150. Immunofluorescence observations using an antibody to the granule protein, eosinophil peroxidase, indicated that the recruited cells had not degranulated. Our results support the importance of IL-5 in the recruitment of tissue eosinophils, but further stimulation is probably required to cause degranulation of these cells and the ensuing tissue damage.**

**Key words:** Allergic inflammation, Eosinophilia, Interleukin 5, Microvascular permeability.

### Introduction

Elevated numbers of eosinophils in the blood and tissues is a common feature associated with allergic reactions.[1–3] Through the generation of inflammatory lipid mediators, like platelet activating factor (PAF) or leukotrienes, and the release of biologically active granule proteins, the eosinophil leukocyte has been documented as a potent effector cell capable of contributing to the pathophysiology of allergic disorders.[4–6] In support of this hypothesis, we and others have shown that eosinophil granule proteins can cause increased vascular macromolecular permeability, which may lead to oedema.[7,8]

Cytokines such as granulocyte-macrophage colony stimulating factor (GM-CSF) and the interleukins (IL) 3 and 5 have been identified as agents that may enhance eosinophil function in allergic inflammatory reactions.[9,10] Recent experiments have documented that IL-5 is the predominant cytokine that can cause the preferential proliferation, differentiation, and tissue recruitment of eosinophil leukocytes.[11,12] Furthermore, IL-5 affects the activation, chemotatic responses, degranulation, and decreased apoptosis of peripheral blood derived eosinophils.[13–15]

Because IL-5 possesses biological activity across species, we used recombinant human IL-5 to induce eosinophilia in the Syrian hamster.[16,17] In order to investigate whether cytokine-induced tissue eosinophilia promotes oedema, we used the plasma clearance of fluoroscein isothiocyanate (FITC)-dextran 150 in the hamster cheek pouch preparation as a measure of changes in vascular permeability.

### Materials and Methods

The male golden Syrian hamsters (80–100 g) used for these experiments were cared for in accordance with the guidelines of the Animal

© 1995 Rapid Science Publishers

Care and Use Committee of both the University of Medicine and Dentistry of New Jersey and Schering–Plough Research Institute, and the NIH Guide for the Care and Use of Laboratory Animals (NIH Publication No. 86–23). Animals were permitted unrestrained access to food and water.

*Enumeration of eosinophils:* In the studies designed to measure eosinophil infiltration into the cheek pouch, hamsters were euthanized by $CO_2$ asphyxiation. The oral surfaces of both cheek pouches were rinsed with normal saline, the membranes were surgically removed, stretched and stapled to a white index card. Mounted membranes were fixed in phosphate buffered formalin (Fisher, Fairlawn, NJ). Using a number 6 ear-punch, sections of cheek pouch membranes were removed and stained for eosinophils using the method of Duffy *et al.*[18] Regions distant from blood vessels were chosen for examination using 100 × magnification. From each tissue ($n = 3$) per treatment group, at least five random fields were chosen using 400 × magnification and the number of eosinophils measured. The data per treatment group were compiled and expressed as the mean number of eosinophils/high power field (#/HPF; $n = 15$).

Eosinophil recruitment and degranulation in the cheek pouch membrane was assessed using an immunofluorescence technique. Membrane tissue samples were cut into 5 μm sections and subsequently stained with rabbit anti-human eosinophil peroxidase (anti-EPO; unpurified antiserum diluted 1:20). This antiserum was developed in our laboratory using purified human eosinophil peroxidase as described previously.[19] Species cross-reactivity with the antiserum was confirmed on hamster eosinophils obtained from peritoneal lavage. No reactivity of these tissues with normal rabbit serum was found (data not shown). Indirect fluorescence microscopy and photography was performed using an affinity purified FITC-labelled goat anti-rabbit IgG (Southern Biotechnology Associates, Birmingham AL). Samples were then stained in haematoxylin and eosin for leukocyte identification.[20] Preparation and analysis were performed without prior knowledge of the treatment applied.

To obtain peritoneal eosinophils, the peritoneum was lavaged with 10 ml of normal saline and the accompanying cells were recovered using a Jelco Teflon catheter (Critikon, Tampa, FL). Cells were centrifuged at 200 × ***g*** for 15 min at 5°C. Cell pellets were then resuspended in 2 ml of phosphate buffered saline (Gibco, Grand Island, NY) with 0.1% foetal calf serum. Slides from cytospin preparations were visualized with Fisher Leukostat stain. A total of 300 cells under 400 × magnification were counted and the differential was recorded.

To evaluate the effect of rHu IL-5 on eosinophil influx into the cheek pouch membrane, hamsters received repetitive injections of 0.01 mg of denatured or native rHu IL-5 in 0.1 ml of saline (Schering–Plough Research Institute, Kenilworth, NJ). The IL-5 was denatured by boiling a 1 mg/ml solution for 5 min, without loss of soluble protein. These injections were given on alternate days with a maximum of three injections per week. This resulted in a total of seven injections of rHu IL-5 for the 18-day period or four injections for the 9-day paradigm. Injections were given subcutaneously to one cheek. Particular care was taken to avoid puncturing the underlying cheek pouch membrane. Treated animals were then returned to their cages.

*Macromolecular clearance and intravital microscopy:* To assess the integrity of the microcirculation following various treatments, separate sets of hamsters were anaesthetized with sodium pentobarbital (60 mg/kg, i.p.) and tracheotomy was performed. The right jugular vein was cannulated for the administration of the fluorochrome tracer and supplemental doses of i.v. anaesthetic. The right carotid artery was cannulated for collection of arterial blood samples. The cheek pouch was prepared according to previously published methods.[8,21,22] Briefly, a two-piece Lucite chamber with a 1-ml reservoir capacity was attached to a single layer of the pouch, delineating a 2.3 $cm^2$ area for intravital microscopy. The chamber reservoir was filled with bicarbonate buffer (131.9 mM NaCl, 4.7 mM KCl, 2.0 mM $CaCl_2$, 1.2 mM $MgSO_4$ and 18.0 mM $NaHCO_3$; pH 7.35) equilibrated with 5% $CO_2$ in 95% $N_2$. Throughout the surgery and experimental protocol, animals were kept on a heating pad to maintain body temperature at 37°C.

Macromolecular clearance from the cheek pouch was measured using the plasma clearance of FITC-dextran (MW 150 000; Sigma Chemical Co., St Louis; FITC-dx 150) as described previously.[8,20] After surgical preparation, the hamster was positioned on a lucite board mounted on the stage of a Nikon Optiphot microscope. A 1 h stabilization period followed the surgery, during which the pouch was continuously suffused with bicarbonate buffer (1 ml/min; 37°C).

FITC-dx 150 was injected as a bolus (100 mg/kg, i.v.) 30 min into the stabilization period. This was followed by the continuous infusion of FITC-dx 150 (0.15 mg/kg/min) for the duration of the study. Blood samples were collected every 30 min from the arterial cannula. In order to determine plasma clearance values, suffusate samples and plasma levels of FITC-dx 150 were determined according to previously published methods.[8] To evaluate the possible direct effects of IL-5 on the microcirculation, rHu IL-5 (0.1 mg/ml, 1 ml total volume) was topically applied to separate cheek pouch preparations as described previously.[8] Briefly, topical application of IL-5 was conducted by interruption of the suffusate, aspiration of the residual bicarbonate buffer, application of the IL-5 solution by a 1 ml syringe to the chamber reservoir, and by incubation for 5 min. All solutions were warmed to 35°C for 5 min prior to application. After application the IL-5 solution was aspirated, the chamber bed washed three times (3 × 1 ml) with bicarbonate buffer (35°C) and the suffusate reestablished.

Intravital microscopy of the cheek pouch membrane was performed using a Nikon Optiphot microscope with 6.3 ×, 10 ×, 20 ×, and 32 × long-working distance Leitz objectives with 10 × Nikon oculars.[8,20,21] The microscope was equipped for transillumination and epi-illumination. Brightfield transillumination was provided by a fibre optic probe inserted into the pouch. The probe was connected to a 100 W halogen lamp. Epi-illumination was provided by a 50 W mercury arc lamp, with appropriate filters for fluorescence. The recording system consisted of a CCD camera (Dage, Michigan City, IN) connected to a Panasonic time generator, a Sony VO 5858 videotape recorder, and a RCA monochrome video monitor. In order to minimize untoward effects from fluorescence epi-illumination, viewing was kept to less than 45 s.[23]

*Statistical analysis:* All values presented are mean ± S.E.M. Statistical significance ($p < 0.05$) was determined using the non-parametric, one-tailed, Mann–Whitney test (Instat™, GraphPad, San Diego, CA).

### Results

*Eosinophil recruitment in cheek pouch membranes and peritoneum:* Hamsters receiving repetitive subcutaneous injections of rHu IL-5 (0.01 mg) to the right cheek showed a substantial increase in the number of eosinophils in the ipsilateral pouch membrane (Fig. 1). A statistically significant rise in the number of membrane eosinophils occurred by the second day. Subsequent injections of rHu IL-5 further increased the number of eosinophils and, by day 18, a 7-fold increase in the number of eosinophils was seen in treated membranes (Fig. 1). In contrast,

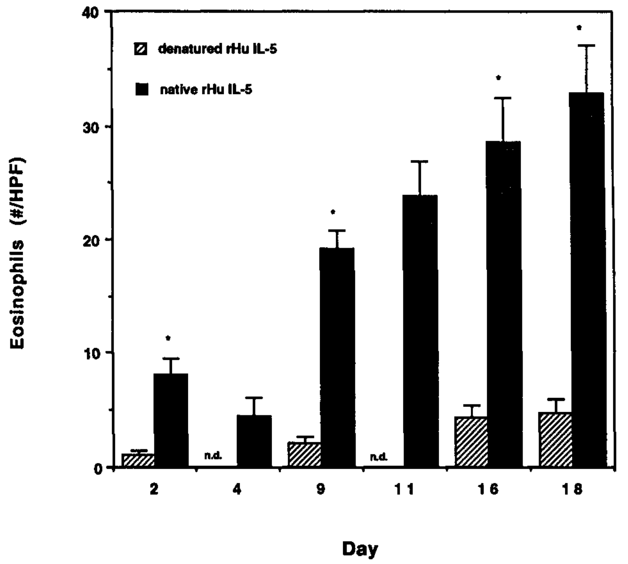

FIG. 1. Time-course of rHu IL-5 induced eosinophilia in the ipsilateral hamster cheek pouch membrane. Animals received repetitive subcutaneous injections of either native or denatured rHu IL-5 as described in Materials and Methods. Membranes were removed, fixed and processed for histological evaluation. Eosinophils were enumerated in five microscopic fields under 400 × magnification. Each treatment group had three animals per day (n.d. = not done). Data reported are the mean eosinophil number per high power field (#/HPF) ± S.E.M. Significance (*) between treatments, within the given day, was determined using the Mann–Whitney non-parametric test, with $p < 0.05$.

animals treated with denatured rHu IL-5 developed a marginal eosinophilia in the ipsilateral cheek pouch membrane only (Fig. 1).

Examination of eosinophil numbers in the contralateral cheek pouch membranes after rHu IL-5 treatment revealed a small but significant increase over 18 days of therapy (Fig. 2). However, eosinophil numbers were considerably less than those in the ipsilateral cheek pouch membrane. There was no significant increase in the recruitment of eosinophils to the contralateral membrane in denatured rHu IL-5 treated animals (Fig. 2).

Using a rabbit anti-Hu EPO antiserum, enumeration of immunopositive cells confirmed the increase in membrane eosinophils by rHu IL-5. Compared to the number of eosinophils that were found in untreated or denatured rHu IL-5 membranes, substantially more cells were detected in the membrane exposed to native rHu IL-5 (Fig. 3a,b,c). Cells reactive with anti-EPO were visible as discrete fluorescent ovals.

Repetitive subcutaneous injections of rHu IL-5 to the cheek pouch of hamsters resulted in a marked increase in the percentage of recoverable peritoneal eosinophils (Fig. 4). Rising from initial values of 12 ± 2.3%, the percentage of peritoneal eosinophils increased to 21 ± 0.9% after 9 days and 34 ± 2.3% after 11 days. Following a similar

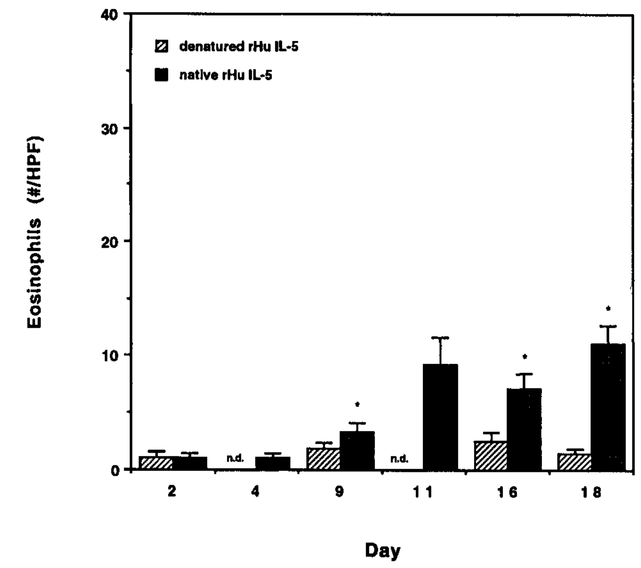

FIG. 2. Time-course of rHu IL-5 induced eosinophilia in the contralateral cheek pouch membrane. Animals received repetitive subcutaneous injections of either native or denatured rHu IL-5 as described in Materials and Methods. The contralateral cheek pouch membrane was removed, fixed and processed for histological evaluation. Eosinophils were enumerated as described in Fig. 1. Data reported are the mean (#/HPF) ± S.E.M. Significance (*; $p < 0.05$) between treatments, for the given day, was determined using the Mann–Whitney non-parametric test.

pattern of administration, denatured IL-5 did not increase the percentage of peritoneal eosinophils (Fig. 4).

*Macromolecular clearance of FITC-dx 150 and intravital microscopy:* To evaluate the acute effects of rHu IL-5, the microcirculation of the hamster cheek pouch was surgically prepared and topically challenged for 5 min with either bicarbonate buffer or rHu IL-5. Compared to bicarbonate buffer, the application of rHu IL-5 (0.1 mg/ml, 1 ml) to the hamster cheek pouch preparation did not result in a significant increase in the clearance of FITC-dx 150. Furthermore, the topical application of rHu IL-5 did not alter vessel diameters, cause muscle fasciculations, or result in demonstrable leukocyte adhesion over the 60 min time-course of experimentation.

Animals were then treated with repetitive subcutaneous injections of rHu IL-5 (0.01 mg/animal; three times per week) to the cheek pouch for a 9-day time-course. During the microsurgical preparation, no distinguishing alterations in the membrane or vascular bed were noted. Furthermore, throughout the stabilization period and the 60 min time-course of the experiment, the microcirculation of the cheek pouch showed no visible fluorescent leakage sites, muscle fasciculations, or substantial alterations in vessel diameter. The suffusate clearance values of FITC-dx 150 obtained from these animals did not deviate significantly from baseline during the 60 min time-course (Fig. 5). Furthermore, the integrated clearance value of FITC-dx 150 obtained from the chronically rHu IL-5 treated animals was not significantly elevated compared to that from cheek pouch preparations exposed to topical rHu IL-5 for 5 min (Fig. 6; $p > 0.05$).

### Discussion

In allergic inflammatory diseases, such as atopic dermatitis and asthma, eosinophils have been hypothesized to contribute to the observed tissue injury. There is increasing evidence that IL-5 is an important mediator for promoting eosinophil responses in these situations. While effecting directed chemotaxis, mature peripheral eosinophils exposed to recombinant IL-5 (rIL-5) show an increase in their respiratory burst, an upregulation of adhesion receptors, augmented degranulatory events and increased *in vitro* survivability.[24–26] Additionally, IL-5 has been documented, *in vitro*, to increase the production of eosinophils from bone marrow cultures, umbilical cord cells, and a number of immortal tumour cell lines.[27–29] Combined, these studies suggest that IL-5 is a late-acting specific factor for eosinophil bone marrow production, release, directed migration and activation.[30]

However, few *in vivo* studies have been conducted where rIL-5 has been directly administered. More often, studies investigating the *in vivo* role of IL-5 on eosinophils have taken the form of administering monoclonal antibodies (mAbs).[31] Through the use of mAbs, researchers have shown that IL-5, exclusively, is important for the eosinophilia that is associated with allergic lung eosinophilia, parasitic infections and episodic angio-oedema.[32,33]

While eosinophils normally populate the hamster cheek pouch membrane, the recruitment and activation of large numbers of eosinophils by the cytokine IL-5 might result in the release of any number of inflammatory agents that induce oedema.[34] This hypothesis is supported by the clinical presentation of capillary leak syndrome in cancer patients undergoing IL-2 treatment. Elevated plasma levels of IL-5 and the activation of eosinophils have been associated with oedema and morbidity in these patients.[35]

Our report conclusively demonstrates that repeated exposure to IL-5 can promote substantial recruitment of eosinophils to the cheek pouch membrane of the hamster. The local and systemic bioactivity of repetitive subcutaneous

(a)

(b)

(c)

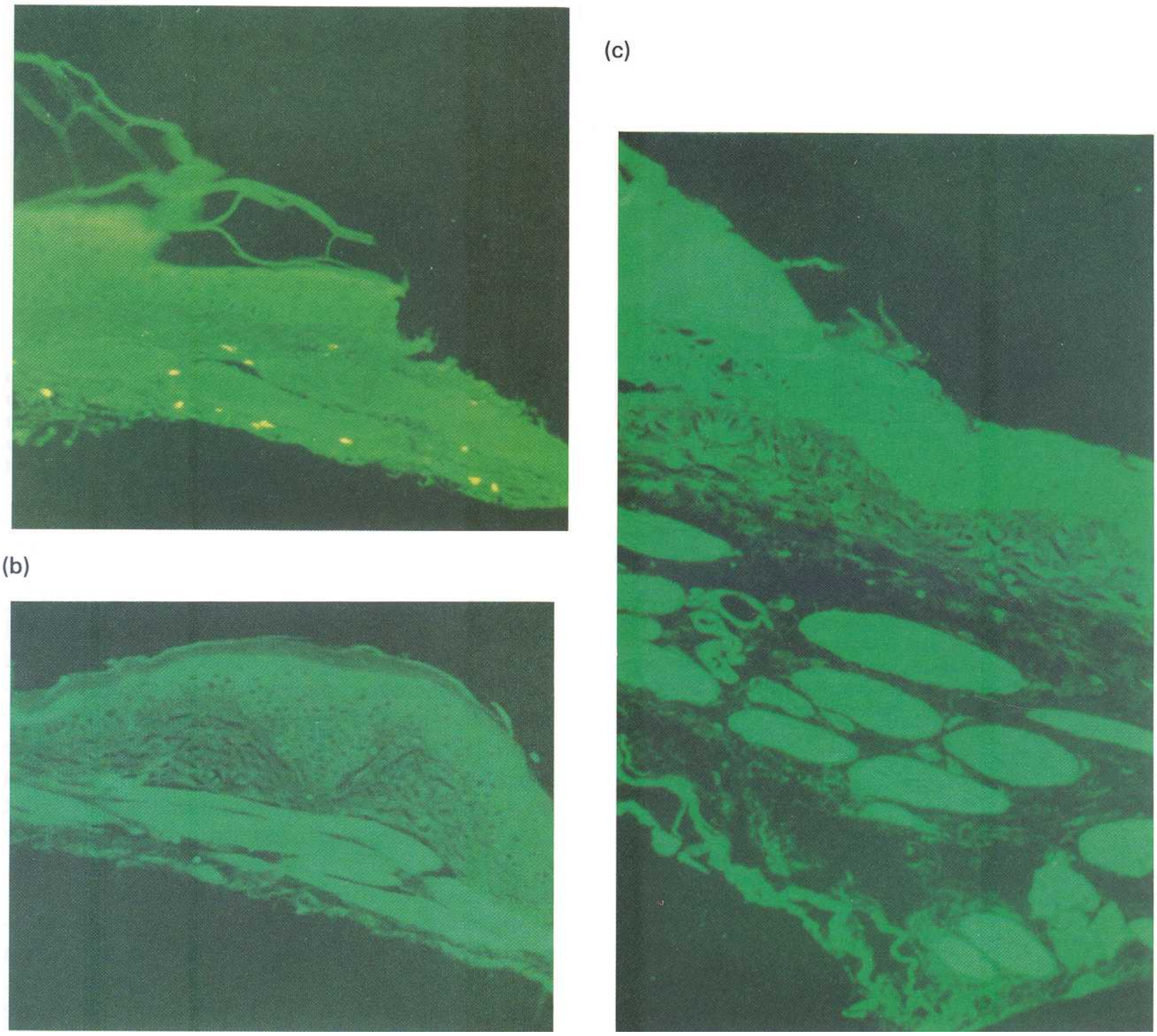

FIG. 3. Immunofluorescent detection of ipsilateral membrane eosinophils. Original representative 18 day samples were at 160× magnification. For publication purposes photomicrographs were reduced. Coded samples from: (a) native rHu IL-5 treated; (b) denatured rHu IL-5 treated; and (c) untreated membranes were incubated with rabbit anti-Hu EPO. Indirect immunofluorescence was conducted using FITC-labelled anti-rabbit IgG. Primary incubation of samples with normal rabbit IgG did not result in fluorescent staining (data not shown).

injections of rHu IL-5 in the hamster is supported by our findings that: (a) histological examination of the underlying ipsilateral pouch membrane revealed a significant increase in the number of eosinophils; (b) less profound eosinophilia occurred in the contralateral pouch membrane; and (c) recoverable cells from peritoneal lavage were enriched in the percentage of eosinophils. However, although evoking a prominent eosinophilia, the repetitive administration of IL-5 did not alter vascular permeability, leading us to conclude that besides IL-5 other factors are required to cause the activation and degranulation of eosinophils under our experimental conditions.

We have previously demonstrated that sub-nanomolar concentrations of the four prominent eosinophil granule proteins: major basic protein (MBP), eosinophil cationic protein (ECP), eosinophil derived neurotoxin (EDN) and eosinophil peroxidase (EPO) have the capacity to increase microvascular permeability in the hamster cheek pouch preparation.[8] Therefore, even partial degranulation of the recruited eosinophils should have resulted in a substantial increase in the clearance of FITC-dx 150. However, our immunofluorescence studies indicated that the recruited eosinophils had not degranulated. Based on the discrete staining pattern with anti-EPO antiserum, this granule protein remained intracellular. Because EPO is found in the matrix of the specific granule, its presence within the cell indi-

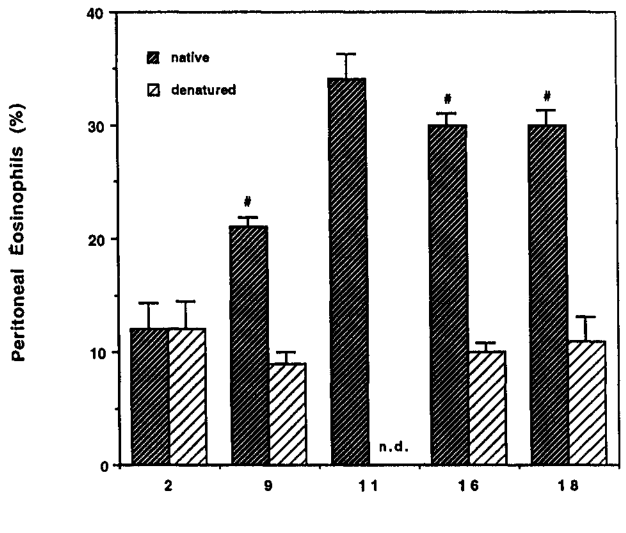

FIG. 4. Effect of rHu IL-5 on peritoneal eosinophils. Animals received repetitive subcutaneous injections to one cheek using denatured or native rHu IL-5 over an 18 day time-course. At appropriate days, the peritoneum was lavaged with saline as described in Materials and Methods. Peritoneal cells were washed and Cytospin preparations were made. At least 300 cells were counted and the percentage of eosinophils was determined. Unless otherwise noted, each day had three animals per treatment (n.d. = not done). Values reported are the mean ± S.E.M. Marginal significance (#; $p = 0.05$) between treatments, for the given day, was determined using the Mann-Whitney non-parametric test.

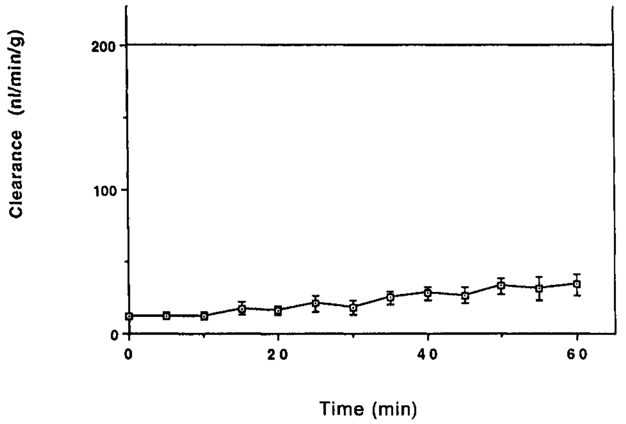

FIG. 5. Effect of rHu IL-5 based eosinophilia on the time-course of clearance of FITC-dx 150. Animals received repetitive injections of rHu IL-5 (0.01 mg per injection) for 9 days. Cheek pouch membranes were arranged for intravital microscopy and the plasma clearance of FITC-dx 150 was monitored for 60 min.

cates that the three remaining proteins-MBP, ECP and EDN were still contained there as well.

It is plausible that the IL-5 induced eosinophilia present in the membranes represents a primed system and subsequent treatment with an appropriate agonist might provoke degranulation of the recruited eosinophils. As a preliminary test of this hypothesis, we treated one of the cheek

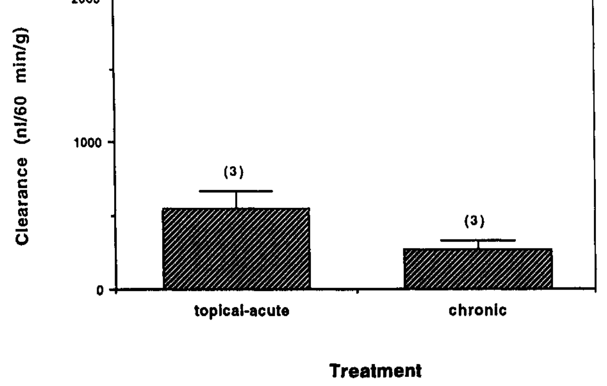

FIG. 6. Comparison of acute and chronic rHu IL-5 administration on the clearance of FITC-dx 150. Cumulative clearance values were determined throughout the 60 min time-course of the experiment in animals receiving rHu IL-5, either 0.1 mg topically for 5 min or 0.01 mg per injection over 9 days. No significant difference between treatment groups was noted ($p > 0.05$).

pouch preparations with a sub-optimal challenge ($10^{-9}$ M) of PAF. In this microcirculatory model, this concentration of PAF is below the threshold needed to increase the clearance of FITC-dx 150.[36,37] Moreover, using isolated human eosinophils, $10^{-9}$ M PAF is a potent degranulatory agent.[38] After challenge, no increase in the clearance of FITC-dx 150 was observed (data not shown), suggesting that the system is not overtly sensitive to stimulation or degranulatory events.

The need for IL-5 bioactivity to cause the eosinophilia was confirmed by treating with denatured rHu IL-5 and failing to find a similar pattern of eosinophilia, both locally and systemically. Nevertheless, at the site of injection, within the underlying membrane, a small but detectable increase in the number of eosinophils was noted. Because no effect was detected in the contralateral membrane or in the peritoneal cell differential, we believe that this small local increase in eosinophils is probably due to the repetitive injections of a foreign denatured protein. The possibility that repetitive needle punctures (27 gauge) may have resulted in a fibrotic wound healing condition, causing the recruitment of eosinophils cannot be excluded.[39] However, we believe this to be an unlikely explanation for our observations. In the wound healing models of tissue eosinophilia, the cutaneous wounds are substantially larger than that provoked by our needle punctures.[39,40] Furthermore, in our model we are studying the underlying oral membrane after the removal of the overlying, loose connective aerolar tissue.

The capacity of IL-5 to direct selective eosinophil chemotaxis has been reported from *in vitro* studies.[41,42] Recent experiments also suggest that local production of IL-5 is responsible for eosinophil recruitment after antigen challenge in allergic individuals.[43] The cellular signaling for eosinophil chemotaxis is through the binding of IL-5 to specific surface receptors on the mature eosinophil leukocyte.[44] In our experimental model, the selected and directed recruitment of the eosinophils might be a result of the cells following a gradient in the administered IL-5, as the molecule was carried out into the systemic circulation. This hypothesis would also partially explain the lack of tissue accumulation of the eosinophils in the IL-5 transgenic animals. In this latter case, IL-5 is constitutively produced by all the T cells of the animal. Therefore, there is no directed tissue recruitment of eosinophils, as there is no specific gradient in IL-5.

In summary, our experiments document that repetitive exposure to rHu IL-5 can selectively recruit eosinophils to the hamster cheek pouch membrane. However, eosinophilia *per se* does not necessarily result in pathological alterations of vascular functions. This lack of pathologic sequelae in response to eosinophilia is consistent with findings in transgenic mice where eosinophilia is not, in general, associated with pathophysiological consequences.[45] However, it is in contrast with the observations of eosinophilia and IL-5 in the IL-2 induced capillary leak syndrome.[35] Because the hamster cheek pouch membrane microcirculation offers an accessible vascular network that has been intensely studied and responds to eosinophil granule proteins, it may be useful for establishing the parameters needed to elicit tissue damage by enhanced eosinophil degranulation.

### References

1. Beasley R, Roche W, Roberts JA, Holgate ST. Cellular events in the bronchi of mild asthma and after bronchial provocation. *Am Rev Respir Dis* 1989; **139:** 806–817.
2. Leiferman KM. Eosinophils in atopic dermatitis. *Allergy* 1989; **44:** 20–26.
3. Rosenberg M. Clinical and immunologic criteria for the diagnosis of allergic bronchopulmonary aspergillosis. *Ann Intern Med* 1977; **86:** 1148–1152.
4. Hodges MK. Heterogeneity of leukotriene C4 production by eosinophils from asthmatic and from normal subjects. *Am Rev Respir Dis* 1988; **138:** 799–804.
5. Lee TC, Lenihan DJ, Malone B, Roddy LL, Wasserman SI. Increased biosynthesis of platelet-activating factor in activated human eosinophils. *J Biol Chem* 1984; **259:** 5526–5530.
6. Hamann KJ, Barker RL, Ten RM, Gleich GJ. The molecular biology of eosinophil granule proteins. *Int Arch Allergy Appl Immunol* 1991; **94:** 202–209.
7. Yoshikawa S, Kayes SG, Parker JC. Eosinophils increase microvascular permeability via the peroxidase–hydrogen peroxide–halide system. *Am Rev Respir Dis* 1993; **147:** 914–920.
8. Minnicozzi M, Durán WN, Gleich GJ, Egan RW. Eosinophil granule proteins increase microvascular macromolecular transport in the hamster cheek pouch. *J Immunol* 1994; **153:** 2664–2670.
9. Weller PF. Cytokine regulation of eosinophil function. *Clin Immunol Immunopath* 1992; **62:** S55–S59.
10. Fujisawa T, Abu-Ghazaleh R, Kita H, Sanderson CJ, Gleich GJ. Regulatory effect of cytokines on eosinophil degranulation. *J Immunol* 1990; **144:** 642–646.
11. Ohnishi T, Kita H, Weiler D, *et al.* IL-5 is the predominant eosinophil-active cytokine in the antigen induced pulmonary late-phase reaction. *Am Rev Respir Dis* 1993; **147:** 901–907.
12. Sanderson CJ. Interleukin 5, eosinophils, and disease. *Blood* 1992; **79:** 3101–3109.
13. Yamaguchi Y, Hayashi Y, Sugama Y, *et al.* Highly purified murine interleukin 5 stimulates eosinophil function and prolongs *in vitro* survival. IL-5 as an eosinophil chemotactic factor. *J Exp Med* 1988; **167:** 1737–1742.
14. Yamaguchi Y, Suda T, Ohta S, Tominaga K, Miura Y, Kasahara T. Analysis of the survival of mature human eosinophils: interleukin-5 prevents apoptosis in mature human eosinophils. *Blood* 1991; **78:** 2542–2547.
15. Kita H, Weiler DA, Abu-Ghazaleh R, Sanderson CJ, Gleich GJ. Release of granule proteins from eosinophils cultured with IL-5. *J Immunol* 1992; **149:** 629–635.
16. Coeffier E, Joseph D, Vargaftig BB. Activation of guinea pig eosinophils by human recombinant IL-5. *J Immunol* 1991; **147:** 2595–2602.
17. Sanderson CJ, Campbell HD, Young IG. Molecular and cellular biology of eosinophil differentiation factor (interleukin 5) and its effects on human and mouse B cells. *Immunol Rev* 1988; **102:** 29–50.
18. Duffy JP, Smith PJ, Crocker J, Matthews HR. Combined staining method for the demonstration of tissue eosinophils and mast cells. *J Histotech* 1993; **16:** 143–144.
19. Abu-Ghazaleh RI, Dunnette SL, Loegering DA, Checkel JL, Kita H, Thomas LL, Gleich GJ. Eosinophil granule proteins in peripheral blood granulocytes. *J Leuk Biol* 1992; **52:** 611–618.
20. Stevens A. The haematoxylins. In: Bancroft JD, Stevens A, eds. *Theory and Practice of Histological Techniques.* New York: Churchill Livingstone. 1990: 107–108.
21. Dillon PK, Durán WN. The effects of platelet activating factor on microvascular permselectivity: dose–response relationships and pathways of action in the hamster cheek pouch microcirculation. *Circ Res* 1988; **62:** 732–740.
22. Mayhan WG, Joyner WL. The effect of altering the external calcium channel blocker, verapamil, on microvascular leaky sites and dextran clearance in the hamster cheek pouch. *Microvasc Res* 1984; **28:** 159–179.
23. Bekker AY, Ritter AB, Durán WN. Reduction of pressure in postcapillary venules induced by epi-fluorescent illumination of FITC-dextran. *Microcirc Endoth Lymphatics* 1987; **3:** 411–423.
24. Lopez AF, Sanderson CJ, Gamble JR, Campbell HD, Young IG, Vadas MA. Recombinant human interleukin 5 is a selective activator of human eosinophil function. *J Exp Med* 1988; **167:** 219–224.
25. Rothenberg ME, Petersen J, Stevens RL, *et al.* IL-5-dependent conversion of normodense human eosinophils to the hypodense phenotype uses 3T3 fibroblasts for enhanced viability, accelerated hypodensity and sustained antibody-dependent cytotoxicity. *J Immunol* 1989; **143:** 2311–2316.
26. Walsh GM, Hartnell A, Wardlaw AJ, Kurihara K, Sanderson CJ, Kay AB. IL-5 enhances the *in vitro* adhesion of human eosinophils, but not neutrophils in a leukocyte integrin (CD11/18)-dependent manner. *Immunol* 190; **71:** 258–265.
27. Fabain I, Lass M, Kletter Y, Golde DW. Differentiation and functional activity of human eosinophilic cells from and eosinophil HL-60 subline: response to recombinant hematopoietic growth factors. *Blood* 1992; **80:** 788–794.
28. Clutterbuck EJ, Hirst EM, Sanderson CJ. Human interleukin 5 (IL-5) regulates the production of eosinophils in human bone marrow cultures: comparison and interactions with IL-1, IL-3, IL-6, and GM–CSF. *Blood* 1989; **73:** 1504–1512.
29. Ishizaka T, Saito H, Katake K, Dvorak AM, Leiferman KM, Arai N, Ishizaka K. Preferential differentiation of inflammatory cells by recombinant human interleukins. *Int Arch Aller Appl Immunol* 1989; **88:** 46–49.
30. McKenzie ANJ, Ely B, Sanderson CJ. Mutated interleukin-5 monomers are biologically inactive. *Mol Immunol* 1991; **28:** 155–158.
31. Gulbenkian AR, Egan RW, Fernandez X, *et al.* Interleukin-5 modulates eosinophil accumulation in allergic guinea pig lung. *Am Rev Respir Dis* 1991; **146:** 263–266.
32. Abrams JS, Van Dyke RE, Gleich GJ. Eosinophil-active cytokines in human disease: development and use of monoclonal antibodies to IL-3, IL-5, and GM-CSF. In: Gleich GJ, Kay AB, eds. *Eosinophils in Allergy and Inflammation.* New York: Marcel Dekker. 1989: 133–157.
33. Coffman RL, Seymour BW, Hudak S, Jackson J, Rennick D. Antibody to interleukin 5 inhibits helminth-induced eosinophilia in mice. *Science* 1989; **245:** 308–310.
34. Ghiabi M, Gallagher GT, Wong DT. Eosinophils, tissue eosinophilia, and eosinophil-derived transforming growth factor alpha in hamster oral carcinoma. *Cancer Res* 1992; **52:** 389–393.
35. van Haelst Pisani C, Kovach JS, Kita H, *et al.* Administration of interleukin-2 (IL-2) results in increased plasma concentrations of IL-5 and the post-treatment eosinophilia in patients with cancer. *Blood* 1991; **78:** 1538–1544.
36. Durán WN, Dillon PK. Acute microcirculatory effects of platelet-activating factor. *J Lipid Med* 1990; **2:** S215–S227.
37. Tomeo A, Egan RW, Durán WN. Priming interactions between platelet activating factor and histamine in the *in vivo* microcirculation. *FASEB J* 1991; **5:** 2850–2855.

38. Kroegel C, Yukawa T, Dent G, Venge P, Chung KF, Barnes PJ. Stimulation of degranulation from human eosinophils by platelet activating factor. *J Immunol* 1989; **142:** 3518–3526.
39. Todd R, Donoff RB, Chiang T, Chou MY, Elovic A, Gallagher G, Wong DTW. The eosinophil as a cellular source of TGF-a in healing cutaneous wound. *Am J Pathol* 1991; **138:** 1307–1313.
40. Wong DTW, Conoff RB, Yang J, *et al.* Sequential expression of transforming growth factors A and B1 by eosinophils during cutaneous wound healing in the hamster. *Am J Pathol* 1993; **143:** 130–142.
41. Wang JM, Rambaldi A, Biondi A, Chen ZG, Sanderson CJ, Mantovani A. Recombinant human interleukin 5 is a selective eosinophil chemoattractant. *Eur J Immunol* 1989; **19:** 701–705.
42. Yamaguchi Y, Hayashi Y, Sugamay Y, *et al.* Highly purified murine interleukin 5 (IL-5) stimulates eosinophil function and prolongs *in vitro* survival. *J Exp Med* 1988; **167:** 1737–1742.
43. Ohnishi T, Sur S, Collins DS, Fish JE, Gleich GJ, Peters SP. Eosinophil survival activity identified as interleukin 5 is associated with eosinophil recruitment and degranulation and lung injury twenty four hours after segmented antigen lung challenge. *J Aller Clin Immunol* 1993; **92:** 607–615.
44. Chihara J, Plumas J, Gruart V, Tavernier J, Prin L, Capion A, Capion M. Characterization of a receptor for interleukin 5 on human eosinophils. *J Exp Med* 1990; **172:** 1347–1351.
45. Dent LA, Strath M, Mellor AL, Sanderson CJ. Eosinophilia in transgenic mice expressing interleukin 5. *J Exp Med* 1990; **172:** 1425–1431.

ACKNOWLEDGEMENT. This work was supported in part by National Institutes of Health Grants HL43146, AI-09728 and AI-34577.

**Received 7 April 1995; accepted in revised form 29 May 1995**

## References

[PDF_00523] Abu-Ghazaleh R. I., Dunnette S. L., Loegering D. A., Checkel J. L., Kita H., Thomas L. L., Gleich G. J. (1992). Eosinophil granule proteins in peripheral blood granulocytes.. J Leukoc Biol.

[PDF_00473] Beasley R., Roche W. R., Roberts J. A., Holgate S. T. (1989). Cellular events in the bronchi in mild asthma and after bronchial provocation.. Am Rev Respir Dis.

[PDF_00536] Bekker A. Y., Ritter A. B., Duran W. N. (1986). Reduction of pressure in postcapillary venules induced by EPI-fluorescent illumination of FITC-dextrans.. Microcirc Endothelium Lymphatics.

[PDF_00608] Chihara J., Plumas J., Gruart V., Tavernier J., Prin L., Capron A., Capron M. (1990). Characterization of a receptor for interleukin 5 on human eosinophils: variable expression and induction by granulocyte/macrophage colony-stimulating factor.. J Exp Med.

[PDF_00555] Clutterbuck E. J., Hirst E. M., Sanderson C. J. (1989). Human interleukin-5 (IL-5) regulates the production of eosinophils in human bone marrow cultures: comparison and interaction with IL-1, IL-3, IL-6, and GMCSF.. Blood.

[PDF_00571] Coffman R. L., Seymour B. W., Hudak S., Jackson J., Rennick D. (1989). Antibody to interleukin-5 inhibits helminth-induced eosinophilia in mice.. Science.

[PDF_00611] Dent L. A., Strath M., Mellor A. L., Sanderson C. J. (1990). Eosinophilia in transgenic mice expressing interleukin 5.. J Exp Med.

[PDF_00529] Dillon P. K., Durán W. N. (1988). Effect of platelet-activating factor on microvascular permselectivity: dose-response relations and pathways of action in the hamster cheek pouch microcirculation.. Circ Res.

[PDF_00581] Durán W. N., Dillon P. K. (1990). Acute microcirculatory effects of platelet-activating factor.. J Lipid Mediat.

[PDF_00551] Fabian I., Lass M., Kletter Y., Golde D. W. (1992). Differentiation and functional activity of human eosinophilic cells from an eosinophil HL-60 subline: response to recombinant hematopoietic growth factors.. Blood.

[PDF_00497] Fujisawa T., Abu-Ghazaleh R., Kita H., Sanderson C. J., Gleich G. J. (1990). Regulatory effect of cytokines on eosinophil degranulation.. J Immunol.

[PDF_00574] Ghiabi M., Gallagher G. T., Wong D. T. (1992). Eosinophils, tissue eosinophilia, and eosinophil-derived transforming growth factor alpha in hamster oral carcinogenesis.. Cancer Res.

[PDF_00564] Gulbenkian A. R., Egan R. W., Fernandez X., Jones H., Kreutner W., Kung T., Payvandi F., Sullivan L., Zurcher J. A., Watnick A. S. (1992). Interleukin-5 modulates eosinophil accumulation in allergic guinea pig lung.. Am Rev Respir Dis.

[PDF_00486] Hamann K. J., Barker R. L., Ten R. M., Gleich G. J. (1991). The molecular biology of eosinophil granule proteins.. Int Arch Allergy Appl Immunol.

[PDF_00480] Hodges M. K., Weller P. F., Gerard N. P., Ackerman S. J., Drazen J. M. (1988). Heterogeneity of leukotriene C4 production by eosinophils from asthmatic and from normal subjects.. Am Rev Respir Dis.

[PDF_00559] Ishizaka T., Saito H., Hatake K., Dvorak A. M., Leiferman K. M., Arai N., Ishizaka K. (1989). Preferential differentiation of inflammatory cells by recombinant human interleukins.. Int Arch Allergy Appl Immunol.

[PDF_00512] Kita H., Weiler D. A., Abu-Ghazaleh R., Sanderson C. J., Gleich G. J. (1992). Release of granule proteins from eosinophils cultured with IL-5.. J Immunol.

[PDF_00483] Lee T., Lenihan D. J., Malone B., Roddy L. L., Wasserman S. I. (1984). Increased biosynthesis of platelet-activating factor in activated human eosinophils.. J Biol Chem.

[PDF_00476] Leiferman K. M. (1989). Eosinophils in atopic dermatitis.. Allergy.

[PDF_00539] Lopez A. F., Sanderson C. J., Gamble J. R., Campbell H. D., Young I. G., Vadas M. A. (1988). Recombinant human interleukin 5 is a selective activator of human eosinophil function.. J Exp Med.

[PDF_00533] Mayhan W. G., Joyner W. L. (1984). The effect of altering the external calcium concentration and a calcium channel blocker, verapamil, on microvascular leaky sites and dextran clearance in the hamster cheek pouch.. Microvasc Res.

[PDF_00562] McKenzie A. N., Ely B., Sanderson C. J. (1991). Mutated interleukin-5 monomers are biologically inactive.. Mol Immunol.

[PDF_00492] Minnicozzi M., Durán W. N., Gleich G. J., Egan R. W. (1994). Eosinophil granule proteins increase microvascular macromolecular transport in the hamster cheek pouch.. J Immunol.

[PDF_00500] Ohnishi T., Kita H., Weiler D., Sur S., Sedgwick J. B., Calhoun W. J., Busse W. W., Abrams J. S., Gleich G. J. (1993). IL-5 is the predominant eosinophil-active cytokine in the antigen-induced pulmonary late-phase reaction.. Am Rev Respir Dis.

[PDF_00603] Ohnishi T., Sur S., Collins D. S., Fish J. E., Gleich G. J., Peters S. P. (1993). Eosinophil survival activity identified as interleukin-5 is associated with eosinophil recruitment and degranulation and lung injury twenty-four hours after segmental antigen lung challenge.. J Allergy Clin Immunol.

[PDF_00542] Rothenberg M. E., Petersen J., Stevens R. L., Silberstein D. S., McKenzie D. T., Austen K. F., Owen W. F. (1989). IL-5-dependent conversion of normodense human eosinophils to the hypodense phenotype uses 3T3 fibroblasts for enhanced viability, accelerated hypodensity, and sustained antibody-dependent cytotoxicity.. J Immunol.

[PDF_00503] Sanderson C. J. (1992). Interleukin-5, eosinophils, and disease.. Blood.

[PDF_00583] Tomeo A. C., Egan R. W., Durán W. N. (1991). Priming interactions between platelet activating factor and histamine in the in vivo microcirculation.. FASEB J.

[PDF_00505] Yamaguchi Y., Hayashi Y., Sugama Y., Miura Y., Kasahara T., Kitamura S., Torisu M., Mita S., Tominaga A., Takatsu K. (1988). Highly purified murine interleukin 5 (IL-5) stimulates eosinophil function and prolongs in vitro survival. IL-5 as an eosinophil chemotactic factor.. J Exp Med.

[PDF_00597] Yamaguchi Y., Hayashi Y., Sugama Y., Miura Y., Kasahara T., Kitamura S., Torisu M., Mita S., Tominaga A., Takatsu K. (1988). Highly purified murine interleukin 5 (IL-5) stimulates eosinophil function and prolongs in vitro survival. IL-5 as an eosinophil chemotactic factor.. J Exp Med.

[PDF_00509] Yamaguchi Y., Suda T., Ohta S., Tominaga K., Miura Y., Kasahara T. (1991). Analysis of the survival of mature human eosinophils: interleukin-5 prevents apoptosis in mature human eosinophils.. Blood.

[PDF_00489] Yoshikawa S., Kayes S. G., Parker J. C. (1993). Eosinophils increase lung microvascular permeability via the peroxidase-hydrogen peroxide-halide system. Bronchoconstriction and vasoconstriction unaffected by eosinophil peroxidase inhibition.. Am Rev Respir Dis.

[PDF_00577] van Haelst Pisani C., Kovach J. S., Kita H., Leiferman K. M., Gleich G. J., Silver J. E., Dennin R., Abrams J. S. (1991). Administration of interleukin-2 (IL-2) results in increased plasma concentrations of IL-5 and eosinophilia in patients with cancer.. Blood.

